# Autophagy Promoted the Degradation of Mutant ATXN3 in Neurally Differentiated Spinocerebellar Ataxia-3 Human Induced Pluripotent Stem Cells

**DOI:** 10.1155/2016/6701793

**Published:** 2016-10-25

**Authors:** Zhanhui Ou, Min Luo, Xiaohua Niu, Yuchang Chen, Yingjun Xie, Wenyin He, Bing Song, Yexing Xian, Di Fan, Shuming OuYang, Xiaofang Sun

**Affiliations:** Key Laboratory for Major Obstetric Diseases of Guangdong Province, Key Laboratory of Reproduction and Genetics of Guangdong Higher Education Institutes, The Third Affiliated Hospital of Guangzhou Medical University, Guangzhou 510150, China

## Abstract

Spinocerebellar ataxia-3 (SCA3) is the most common dominant inherited ataxia worldwide and is caused by an unstable CAG trinucleotide expansion mutation within the* ATXN3* gene, resulting in an expanded polyglutamine tract within the ATXN3 protein. Many* in vitro *studies have examined the role of autophagy in neurodegenerative disorders, including SCA3, using transfection models with expression of pathogenic proteins in normal cells. In the current study, we aimed to develop an improved model for studying SCA3* in vitro *using patient-derived cells. The patient-derived iPS cells presented a phenotype similar to that of human embryonic stem cells and could be differentiated into neurons. Additionally, these cells expressed abnormal ATXN3 protein without changes in the CAG repeat length during culture for at least 35 passages as iPS cells, up to 3 passages as neural stem cells, and after 4 weeks of neural differentiation. Furthermore, we demonstrated that neural differentiation in these iPS cells was accompanied by autophagy and that rapamycin promoted autophagy through degradation of mutant ATXN3 proteins in neurally differentiated spinocerebellar ataxia-3 human induced pluripotent stem cells (*p* < 0.05). In conclusion, patient-derived iPS cells are a good model for studying the mechanisms of SCA3 and may provide a tool for drug discovery* in vitro*.

## 1. Introduction

Spinocerebellar ataxia-3 (SCA3) is one of nine polyglutamine (polyQ) disorders caused by a CAG expansion mutation within the* ATXN3* gene, which encodes the ATXN3 protein [1]. Expansion of the polyQ tract results in neuronal cytotoxicity, with calpain-dependent proteolysis of the mutant ATXN3, thereby generating expanded polyQ fragments and insoluble aggregates and leading to the formation of inclusions in the nucleus and cytoplasm of neurons, including axonal tracts [2, 3]. The onset of SCA3 occurs during adulthood and is associated with gait and stance abnormalities, limb ataxia, dysarthria, dysphagia, oculomotor dysfunction, pyramidal and extrapyramidal signs, peripheral neuropathy, and aspiration pneumonia resulting from dysphagia [4]. The pathogenic mechanisms of SCA3 and other polyQ disorders are not well understood, and there are currently no effective cures for these disorders.

Cells continuously turn over proteins through cycles of synthesis and degradation to maintain cellular homeostasis. The ubiquitin-proteasome system is a process for selective degradation of proteins, and recent studies have highlighted the role of lysosomes in this selective degradation process via autophagy [5]. Autophagy is a highly conserved biological process involving bulk degradation of proteins and organelles, during which portions of the cytoplasm are sequestered into double-membrane vesicles known as autophagosomes. These autophagosomes then fuse with lysosomes to form single-membrane autolysosomes; ultimately, the contents of autolysosomes are degraded by lysosomal hydrolases and recycled for energy utilization. This process helps to maintain cellular homeostasis and protects organisms from damage and diseases [6]. Dysregulation of autophagy is involved in many human diseases, including cancer, infection, immunity, heart disease, liver disease, aging, myopathies, and neurodegeneration [7]. Alzheimer's disease (AD), Parkinson's disease (PD), Huntington's disease (HD), and dominant spinocerebellar ataxia (SCA) are the most common neurodegenerative diseases exhibiting accumulation of abnormal protein aggregates. Several studies have demonstrated that inhibition of mammalian target of rapamycin (mTOR) complex 1 (mTORC1) by rapamycin promotes the degradation of mutant proteins* in vitro* and reduces the severity of neurodegeneration in animal models [8–10].

Induced pluripotent stem (iPS) cells, which are generated by transduction with a set of transcript factors in human somatic cells, offer an alternative cellular model for mechanistic studies [11]. Patient-derived iPS cells can preserve the genetic mutations carried by the patient in a functional human genomic background. Furthermore, these cells can be differentiated into human cells of neural lineage, which could be advantageous for investigating the pathogenic mechanisms of a disease and identifying potential therapies.

In this study, we used an episomal reprogramming assay to produce SCA3-iPS cells from skin fibroblasts of a woman with SCA3. We then examined the effects of autophagy during the neural differentiation of SCA3-iPS cells. Our results suggested that patient-specific iPS cells may represent an effective model system for analysis of potential therapies in a variety of diseases, including SCA3.

## 2. Materials and Methods

### 2.1. Cell Culture

The study was performed in accordance with the Declaration of Helsinki and has been approved by the ethical committee of The Third Affiliated Hospital of Guangzhou Medical University. Informed consent was obtained from all patients. hESC line 10 (hES-10), which was used as a positive control, was established in our hospital [12]. SCA3-iPS cells were generated using episomal reprogramming assays (Invitrogen, Carlsbad, CA, USA) from a woman (skin fibroblasts) with SCA3 who suffered from gait and stance abnormalities, limb ataxia, dysarthria, dysphagia, and oculomotor dysfunctions and had 81 CAG repeats in the* ATXN3* gene, as determined using polymerase chain reaction (PCR), fragment analysis, and sequencing. Two clones from the patient (SCA3-iPS-1 and SCA3-iPS-2) were used for the experiments. Cell lines were cultured using Essential 8 Medium (Gibco, USA)/Geltrex LDEV-Free hESC-qualified Reduced Growth Factor Basement Membrane Matrix (Invitrogen) in a feeder-independent culture system, as previously described [13].

### 2.2. CAG Repeats Length Analysis

The primers used for detection of the CAG repeat in the* ATXN3* gene by PCR and fragment analysis were as follows: Fam-CAGTGACTACTTTGATTCG and TGGCCTTTCACATGGATGTGAA. The primers for sequencing were described previously [3].

### 2.3. Stem Cell and Pluripotency Analysis

Reverse-transcription PCR (RT-PCR) was performed to detect the expression of endogenous pluripotency genes according to the manufacturer's instructions (TaKaRa, Japan). The primers were as follows:* Oct4*, 5′-GACAGGGGGAGGGGAGGAGCTAGG-3′ and 5′-CTTCCCTCCAACCAGTTGCCCCAAAC-3′; Nanog, 5′-CAGCCCCGATTCTTCCACCAGTCC-3′ and 5′-CGGAAGATTCCCAGTCGGGTTCACC-3′. AP staining and immunofluorescence analysis were performed as previously described [14]. The primary antibodies used were as follows: anti-SSEA-3 (1 : 100; Sigma, USA), anti-TRA1-60 (1 : 200; Sigma), anti-TRA1-81 (1 : 500; Sigma), anti-alpha-fetoprotein (AFP; 1 : 500; Sigma), anti-Nestin (1 : 100; Abcam, England), anti-PAX6 (1 : 200; Sigma), anti-NeuN (1 : 100; Abcam), beta-tubulin III (1 : 100; Abcam), and anti-smooth muscle actin (SMA; 1 : 500; Sigma).

### 2.4. *In Vivo* and* In Vitro* Differentiation

The colonies were harvested and subcutaneously injected into the inguinal grooves of 6-week-old male mice with severe combined immunodeficiency (SCID) as previously described [15]. Embryoid body (EB) formations were performed as previously described [16].

### 2.5. Karyotype Analysis

After Giemsa staining, at least 20 cells were examined in each group for chromosome analysis as previously described [14].

### 2.6. Neural Stem Cells (NSCs) and Neural Differentiation

NSCs were cultured using Gibco PSC neural induction medium and StemPro NSC SFM (Life Technologies, USA), and neural differentiation was performed according to the manufacturer's instructions. Briefly, about 24 h after hESCs and iPS cells were split into six-well plates, the culture medium was switched to Gibco PSC neural induction medium containing neurobasal medium and Gibco PSC neural induction supplement. The neural induction medium was changed every other day from the beginning of neural induction. The neural induction medium was changed every day after 4 days of neural induction. At day 7, primitive NSCs were dissociated using Accutase (Life Technologies) and plated on Geltrex-coated dishes in an NSC expansion medium containing 50% neurobasal medium, 50% Advanced DMEM/F12, and neural induction supplement. The NSC expansion medium was changed every other day until NSCs reached confluence at day 5 after plating of primitive NSCs [17]. For neural differentiation, NSCs were plated onto laminin (10 *μ*g/mL; Life Technologies) coated six-well chamber slides at a density of 5 × 10^4^ cells/cm^2^ in a neuronal differentiation medium consisting of neurobasal medium, B-27, and GlutaMAX (Life Technologies). The culture medium was changed every 2-3 days, according to the manufacturer's instructions (Life Technologies).

### 2.7. Western Blot Analysis

The protein content was determined using a BCA protein assay kit (Thermo Fisher) according to the manufacturer's instructions. Equivalent amounts of protein were separated on 10–15% sodium dodecyl sulfate- (SDS-) polyacrylamide gels and blotted onto nitrocellulose membranes. After being blocked at room temperature for 2 h with 5% nonfat milk in TBS with 0.1% Tween-20, the membranes were probed with anti-LC3B (1 : 1000; Cell Signaling Technology, USA), anti-p62 (1 : 1000; Abcam), anti-GAPDH (1 : 5000; Cell Signaling Technology), anti-ATXN3 (1 : 1000; Abcam), and horseradish peroxidase- (HRP-) conjugated IgG antibodies (1 : 10000; Cell Signaling Technology). The volumes of the bands were determined by standard scanning densitometry with normalization of densitometry measures to the expression of GAPDH.

### 2.8. Flow Cytometry Assay

Single cells derived from NSCs were fixed and permeabilized following the manufacturer's instructions (BD Pharmingen, USA). Cells were then stained with the following monoclonal fluorochrome-conjugated antibodies: Alexa Fluor® 647 mouse anti-NESTIN (BD Biosciences, USA) and Alexa Fluor® 488 mouse anti-human PAX6 (BD Biosciences, USA). Flow cytometry was performed using FACSAria™III flow cytometer (BD Biosciences, USA).

### 2.9. Chromosomal Microarray Analysis–Single Nucleotide Polymorphism Array Analysis

Chromosomal microarray analysis–single nucleotide polymorphism array analysis was performed for a higher resolution. DNA was prepared and hybridized to the CytoScan HD array (Affymetrix) platform according to the manufacturer's protocol [18].

### 2.10. Statistical Analysis

Data are expressed as the mean ± standard error of the mean (SEM) and were compared by one-way analysis of variance (ANOVA). When ANOVA results were significant, differences between groups were assessed by* post hoc* testing using LSD tests (SPSS version 17.0 for Windows). Differences with *p* < 0.05 were considered statistically significant.

## 3. Results

### 3.1. Characterization of SCA3-iPS Cells

**Figure 1:**
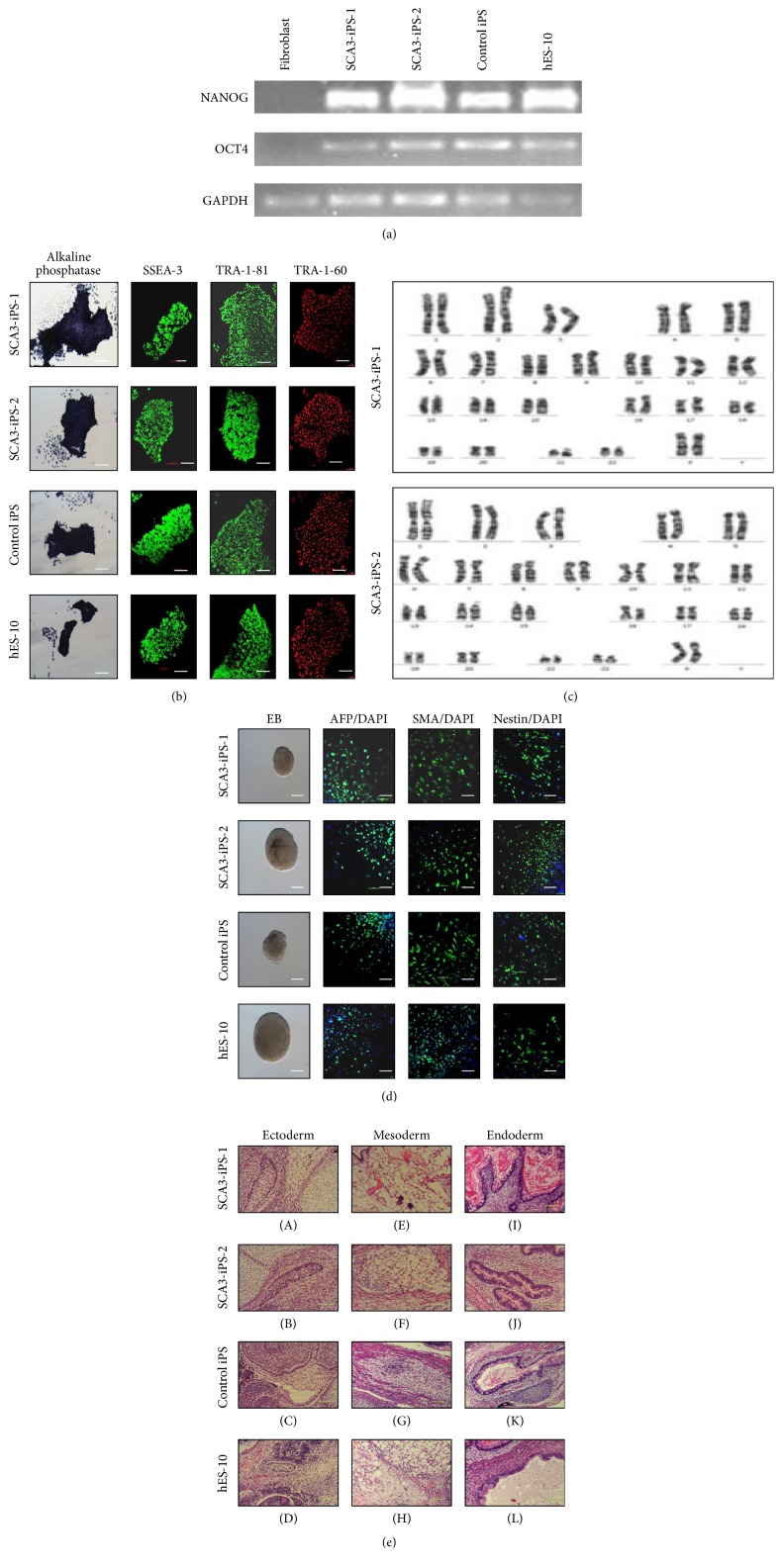
Characterization of SCA3-iPS cells. (a) RT-PCR analysis of the expression of undifferentiated hES cell marker genes in SCA3-iPS cells. (b) Immunostaining of iPS cells for cell surface markers, including alkaline phosphatase, SSEA-3, TRA-1-60, and TRA-1-81. Scale bars, 100 *μ*m. (c) Karyotype of SCA3-iPS cells. (d) Immunofluorescence results showed differentiated SCA3-iPS cells expressing AFP (endoderm marker), SMA (mesoderm marker), and Nestin (ectoderm marker). Scale bars, 100 *μ*m. (e) Teratomas contained tissues from all three types of germ layers, including ectoderm, that is, sebaceous glands (A, B, and C) and neural canal (D); mesoderm, that is, adipose tissue (E, F, and H) and smooth muscle (G); and endoderm, that is, glandular tissue (I, J, K, and L). Cell nuclei were stained with DAPI (blue). Scale bars, 20 *μ*m.

RT-PCR analysis showed that the cells were positive for endogenous expression of* Oct4* and* Nanog* (Figure 1(a)). Immunofluorescence analysis of stem cell markers in SCA3 and control iPS colonies indicated positive staining for AP, SSEA-3, TRA-1-60, and TRA-1-81 (Figure 1(b)). Karyotyping analysis showed a normal female karyotype (Figure 1(c)).

### 3.2. Differentiation* In Vitro* and* In Vivo*

EBs were used to determine the differentiation ability of SCA3-iPS cells* in vitro*. Immunofluorescence results showed that the differentiated cells were positive for AFP (endoderm), SMA (mesoderm), and Nestin (ectoderm; Figure 1(d)). To demonstrate the pluripotency of SCA3-iPS cells* in vivo*, SCA3-iPS cells were subcutaneously injected into SCID mice. Eight weeks after injection, we observed teratoma formation. Histological examinations showed that the teratomas contained various tissues comprising all three germ layers (Figure 1(e)).

### 3.3. SCA3-iPS Cells Could Differentiate into NSCs and Neurons

**Figure 2:**
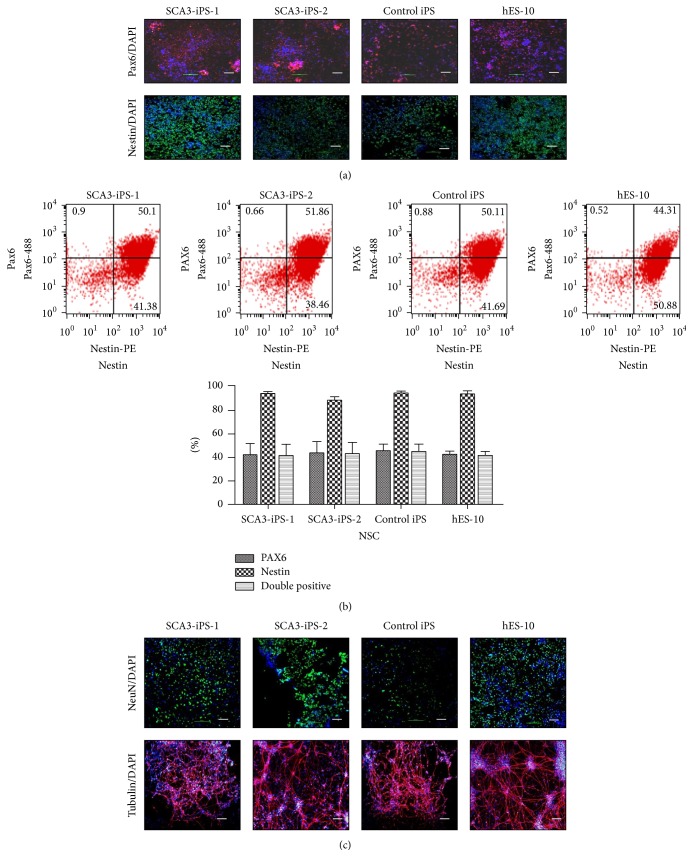
SCA3-iPS cells could differentiate into NSCs and neurons. (a) Immunofluorescence results for Pax6 and Nestin. (b) The flow cytometry analysis below showed that no significant difference was observed in the percentage of Pax6 or Nestin positive cells or Pax6 and Nestin-double positive cells (*n* = 3). (c) Immunofluorescence results for NeuN and beta-tubulin III. Cell nuclei were stained with DAPI (blue). Scale bars, 20 *μ*m.

Both SCA3-iPS cells (15–20 passages) and normal control iPS cells (15–20 passages) could be differentiated into NSCs and neurons. Approximately 24 h after passaging, the culture medium was switched to Gibco PSC neural induction medium containing neurobasal medium and Gibco PSC neural induction supplement. At day 7 of induction, primitive NSCs were formed, and immunofluorescence results showed that cells were positive for Pax6 and Nestin, which are NSC markers (Figure 2(a)). And the flow cytometry result showed no difference in these markers (Figure 2(b)). After NSCs expansion for several passages, neural induction medium was added for neural differentiation. Around day 21, immunofluorescence for NeuN and tubulin confirmed their neural lineage (Figure 2(c)).

### 3.4. CAG Repeats and ATXN3 Protein Remained Stable during Reprogramming and Neural Differentiation

**Figure 3:**
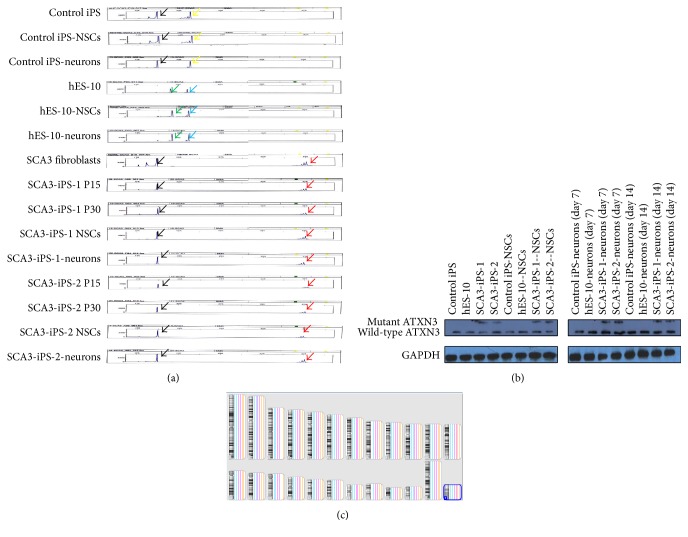
CAG repeats, genome, and mutant ATXN3 protein remained stable during reprogramming and neural differentiation. (a) Repeat sizes were confirmed by PCR with fluorescently labeled primers and capillary electrophoresis. The expanded alleles showed no detectable differences (15 (black arrows) and 81 (red arrows)) in fibroblasts (P3), SCA3 iPS cells (P15 and P30), NSCs (P3), and neurons (day 28) (yellow arrows: 30 repeats; green arrows: 23 repeats; blue arrows: 29 repeats). (b) Western blot bands from SCA3-iPS cells, NSCs, neurons, and control cells. P = passage. (c) No obvious* de novo* mutations in the fibroblasts (green), iPS cells (yellow), and the neurons (pink).

Expansion of the CAG repeats in the* ATXN3 *gene was verified by PCR and fragment analysis in fibroblasts (passage 3), SCA3-iPS cells (passages 15 and 30), NSCs (passage 3), and neurons (28 days). All cells showed stable CAG repeats (15 and 81; Figure 3(a)). Western blots were performed to detect the protein expression of ATXN3 in iPS cells, NSCs, and neurons derived from the patient with SCA3. Specific bands around 42 kDa (ATXN3) and 60 kDa (mutant ATXN3) were detected in all cell lines derived from the patient with SCA3, while control iPS cells and hES-10 had only the normal band of nonexpanded ATXN3 at around 42 kDa (Figure 3(b)).

### 3.5. No Obvious* De Novo* Mutations in Genome during Reprogram and Differentiation

To detect deletions or duplication across the whole genome, a chromosomal microarray analysis–single nucleotide polymorphism analysis was performed in the fibroblasts, iPS cells, and the neurons. In accordance with the karyotyping results, neither* de novo* deletions nor duplications were found in these cells.

### 3.6. Involvement of Autophagy during Neural Differentiation

**Figure 4:**
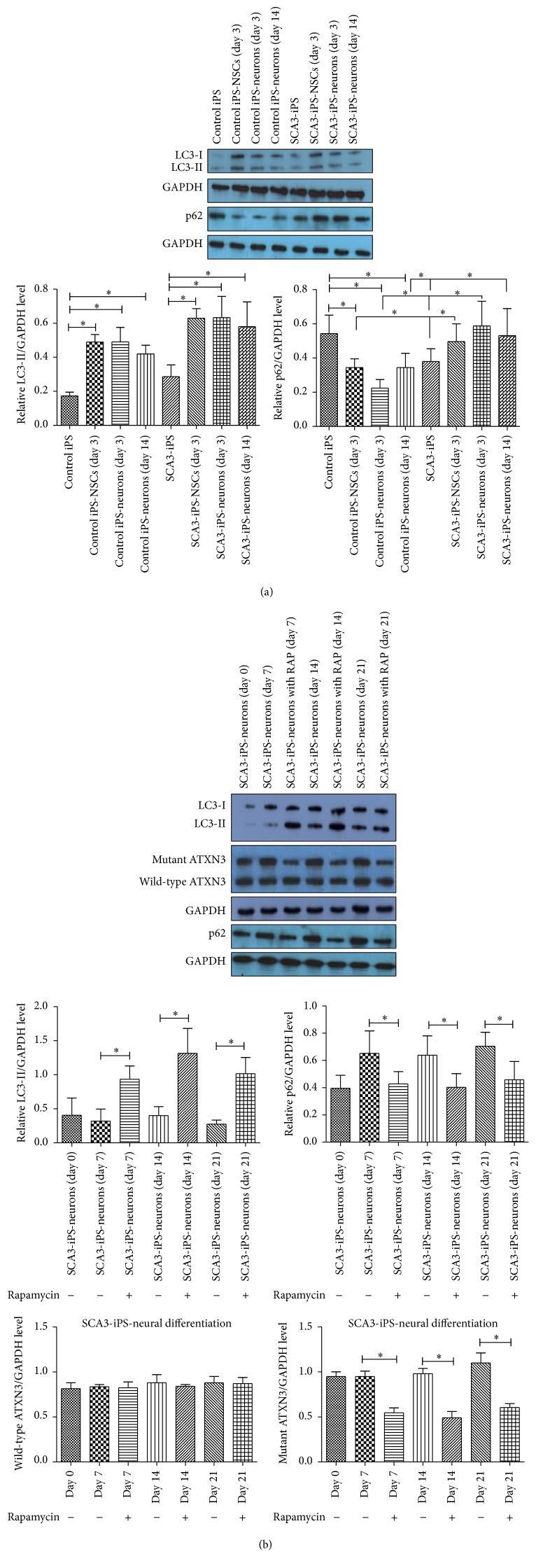
Autophagy was increased during neural differentiation and autophagy promoted the degradation of mutant ATXN3 in neurally differentiated spinocerebellar ataxia-3 human iPS cells. (a) Western blot was used to determine the levels of LC3-II and P62 in various cell types on days 3 and 14. Quantification of the band intensity is shown in the lower panel. (b) Western blot analysis was performed on days 0, 7, 14, and 21. Quantification of the band intensity is shown in the lower panel. ^*∗*^ *p* < 0.05 (*n* = 3).

Anti-LC3B antibodies were used to detect the level of autophagy in iPS cells and NSCs (day 3) and during neural differentiation (days 3 and 14) of control iPS cells and SCA3-iPS cells. The results showed that autophagy was increased during neural differentiation (*p* < 0.05). And the levels of LC3-II were increased during the SCA3 neural differentiation compared with the control iPS neural differentiation, but the increase levels were not of significant difference (*p* > 0.05). However, the levels of p62 were increased during the SCA3 neural differentiation compared with the control iPS neural differentiation (*p* < 0.05); these results may indicate impairment in the trafficking to lysosomes and protein degradation (Figure 4(a)).

### 3.7. Autophagy Promoted the Degradation of Mutant ATXN3 in Neurally Differentiated Spinocerebellar Ataxia-3 Human iPS Cells

Beginning on the first day of neural differentiation, we added 100 nM rapamycin (an autophagy inducer; Sigma) to the neural differentiation medium. The culture medium (with 20 nM rapamycin) was changed every 2-3 days. Cells were harvested, and western blot analysis was performed on days 0, 7, 14, and 21. Levels of LC3-II protein were increased following rapamycin treatment compared with that in untreated cells (*p* < 0.05), and levels of p62 protein were decreased (*p* < 0.05), indicating upregulation of autophagy. Moreover, the levels of the expanded polyQ ATXN3 protein (60 kDa) were significantly reduced (*p* < 0.05); however, there were no obvious changes in the levels of the wild-type ATXN3 protein (42 kDa; *p* > 0.05). These results suggested that the mutant protein was more efficiently degraded by autophagy than the wild-type protein (Figure 4(b)).

## 4. Discussion

SCA3 is characterized by the formation of intraneuronal inclusions, particularly in brain regions such as the cerebellum, substantia nigra, and pontine nuclei [19]. However, the mechanism underlying the formation of these inclusions is still poorly understood because of the lack of an appropriate model [10, 20]. Recent studies reported a novel mouse model of SCA3 that could be used to study the pathogenesis and treatment of SCA3. However, there are many differences between mice and humans, and a mouse model may not be completely applicable to humans. Patient-derived iPS cells preserve the genetic mutation carried by the patient on a functional human genomic background and can be differentiated into human cells of a neural lineage. Thus, this feature may be advantageous for investigating the pathogenic mechanisms of SCA3 and for the development of appropriate therapies to treat the disease. SCA3-iPS cell line could differentiate into human cells of neural lineage. Additionally, CAG repeats remained stable during reprogramming and neural differentiation, and the abnormal ATXN3 protein was expressed in SCA3-iPS cells and neurons. Furthermore, the chromosomal microarray analysis–single nucleotide polymorphism analysis showed no obvious* de novo* mutations in whole genome during reprogram and differentiation.

In humans with polyQ expansion disorders, expanded alleles are prone to changes in repeat length. These changes are related to both meiosis and mitosis in male gametes [21]. In our SCA3 iPS cells, both the expanded allele and normal allele remained stable with regard to repeat length during reprogramming, proliferation, and differentiation from iPS cells to neurons, consistent with a previous study by Koch and Xia [3, 22]. We assumed that the stable CAG repeats observed in our cell line may be explained by genomic stability during reprogramming, proliferation, and differentiation.

Autophagy is a bulk lysosomal degradation pathway involved in recycling of long-lived proteins and cytoplasmic organelles for cell survival [23, 24]. On induction of autophagy, the conversion of LC3-I into LC3-II is indicative of autophagosome formation and is therefore widely used as a marker for autophagosome formation. In many types of tissues, basal autophagy is induced by starvation; in neurons, autophagy is also constitutively active at a low level [25]. Moreover, autophagy is critical for stem cell reprogramming, renewal, proliferation, and differentiation [26–29]. In our results, we also found that basal autophagy was present in control iPS neuronal differentiation. Interestingly, autophagy is typically upregulated in various HD mouse models and in neuronal and nonneuronal cells from patients with HD [30, 31]. Besides, another study reported that accumulation of p62 and light chain 3-positive autophagosomes has been associated with impairment in the trafficking to lysosomes and protein degradation in the putamen of the SCA3 patient [32]. These results were consistent with our* in vitro* neuronal differentiation of SCA3-iPS cells. Growing evidence has revealed that regulation of autophagy is involved in many human diseases, including neurodegeneration [8, 20]. Aggregation of the mutated form of the disease protein ATXN3 into neuronal nuclear inclusions has been studied extensively in SCA3. The misfolded proteins in cells could be initially cleared by the molecular chaperone and the ubiquitin-proteasome system [33]. When these systems malfunction, the misfolded proteins accumulate and form oligomers and then small aggregates, which are potentially cytotoxic [34]. Several studies have demonstrated that the inhibition of mTORC1 by rapamycin promotes the degradation of mutant proteins* in vitro* and results in a reduction in the severity of neurodegeneration in several models* in vivo *[10, 20, 35]. Studies have also shown that rapamycin plays a role in neuroprotection in a Parkinson's disease murine model by blocking mTORC1-dependent translation of the pro-cell death protein RTP801 [36]. However, many* in vitro* studies investigating the function of autophagy in neurodegenerative disorders have been based on transfection with genes encoding pathogenic proteins in normal cells [8]. iPS cells generated from SCA3-patient and retaining the disease-causing ATXN3 mutation have been reported by Koch and Hansen, but they did not study the relation between ATXN3 proteins and autophagy [3, 37]. In the present study, we used iPS cells induced from somatic cells of a patient with SCA3. Rapamycin was also added to the neural differentiation medium to promote autophagy to degrade mutant proteins in neurally differentiated spinocerebellar ataxia-3 human iPS cells. The results showed that the amount of mutant ATXN3 protein (about 60 kDa) of rapamycin-treated neuronal differentiation was lower than that without rapamycin treatment, demonstrating that rapamycin-dependent autophagy could reduce the levels of mutant proteins. However, there were no significant effects on wild-type ATXN3. These results were consistent with previous studies suggesting that mutant proteins depend more on autophagy for clearance [38]. Notably, we treated the cells with rapamycin early for the following reasons. First, neurons are more prone to accumulate cytotoxic proteins than other cell types because they cannot dilute toxic substances by means of cell division [39]. Moreover, mutant proteins need to be packaged into autophagic vacuoles in dendrites and axons and then be retrogradely transported to the cell body for degradation [40]. Additionally, young neurons can clear cytotoxic proteins; however, this process becomes increasingly difficult as neurons age because of the downregulation of chaperone-mediated autophagy and macroautophagy at the transcriptional, translational, and posttranslational levels [25, 41]. Lastly, rapamycin may not be effective during the late stage of these diseases. Therefore, early upregulation of autophagy may have therapeutic potential in treating SCA3. However, autophagy can only eliminate cytoplasmic mutant proteins; hence, the cellular localization of mutant proteins is a key factor when targeting upregulation of autophagy as a therapeutic strategy for their elimination. Different neurodegenerative diseases show different distributions of these aggregates. For example, in spinal and bulbar muscular atrophy SCA1, SCA7, and SCA17, proteins accumulate in the nucleus, whereas, in SCA2 and SCA6, proteins accumulate in the cytoplasm, and, in SCA3 and HD, proteins accumulate in both the nucleus and cytoplasm [42].

## 5. Conclusion

In summary, we found that SCA3-iPS cells could be differentiated into neurons to maintain patient-specific genome information. We also demonstrated that neural differentiation was accompanied by autophagy and that rapamycin promoted autophagy, which resulted in degradation of mutant ATXN3 in neurally differentiated spinocerebellar ataxia-3 human iPS cells. Thus, patient-derived iPS cells are a good model for studying the mechanisms of SCA3 and may provide a powerful tool for drug discovery.
